# Endoscopic Nasogallbladder Drainage Combined with Laparoscopic Surgery for Type I Mirizzi Syndrome with Acute Cholecystitis: A Case Series Report

**DOI:** 10.1155/2020/2417539

**Published:** 2020-04-06

**Authors:** Wei Han, Qing Yue, Kai Liu, Jian-ji Ke, Ling-yu Meng, Ya-hui Liu

**Affiliations:** ^1^Department of Hepatobiliary and Pancreatic Surgery, The First Hospital of Jilin University, No. 71 Xinmin Street, Changchun, 130021 Jilin, China; ^2^Department of Oncology, The First Hospital of Jilin University, No. 71 Xinmin Street, Changchun, 130021 Jilin, China

## Abstract

**Objective:**

To investigate the safety and feasibility of endoscopic nasogallbladder drainage (ENGBD) combined with laparoscopic surgery for Mirizzi syndrome type I with acute cholecystitis.

**Methods:**

An analysis of 4 patients with type I Mirizzi syndrome with acute cholecystitis admitted to the First Hospital of Jilin University.

**Results:**

The patients underwent ENGBD, and laparoscopic surgery was evaluated postoperatively. All four patients successfully recovered from this combined surgical approach.

**Conclusion:**

The combination of ENGBD and laparoscopic surgery is safe and feasible for the treatment of patients with type I Mirizzi syndrome accompanied by acute cholecystitis. This approach may reduce the traumatic stress on patients and is worthy of widespread implementation.

## 1. Introduction

Mirizzi syndrome (MS) is characterized by obstructive jaundice and cholangitis caused by stones in the cystic duct and gallbladder neck. MS also involves inflammation of the common bile duct, caused by the pressure associated with cholelithiasis (1). Because of the complexity of these pathological changes, surgical treatment for MS is difficult to perform and is associated with high risk for complications.

In recent years, endoscopic retrograde cholangiopancreatography (ERCP) and endoscopic nasobiliary drainage (ENBD) have been used in combination to treat MS. The effectiveness and safety of this combined approach have rendered it the gold standard for the treatment of MS (2). We honed this technique at the First Hospital of Jilin University, using ERCP and endoscopic nasogallbladder drainage (ENGBD) combined with laparoscopic surgery for MS type I (according to Csendes' classification) with acute cholecystitis (3).

The objective of this report was to investigate the safety and feasibility of ENGBD combined with laparoscopic surgery for MS type I with acute cholecystitis. The present report was exempted from the ethical review by the Jilin University Ethics Review Board.

## 2. Materials and Methods

All four patients were treated in the Department of Hepatobiliary and Pancreatic Surgery at the First Hospital of Jilin University during the period from May 2017 to December 2017. All four patients immediately underwent a routine blood and liver function tests, as well as color Doppler ultrasound examination, computed tomography (CT), and magnetic resonance cholangiopancreatography (MRCP) after admission to the emergency department (see details in Table 1). A detailed medical history was required. The diagnosis was based on the patient's medical history, the results of the physical examination, and the results of laboratory testing.

**Figure 1:**
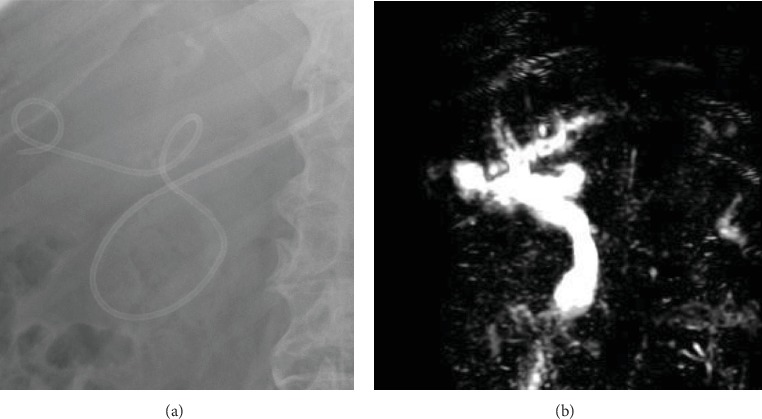
Images of Case 2. (a) X-ray image showing successful placement of a curved nasobiliary duct into the gallbladder cavity. (b) Magnetic resonance cholangiopancreatography (MRCP) image showing that gallbladder volume was reduced postoperatively, with a significant decrease in compression of the common bile duct.

**Figure 2:**
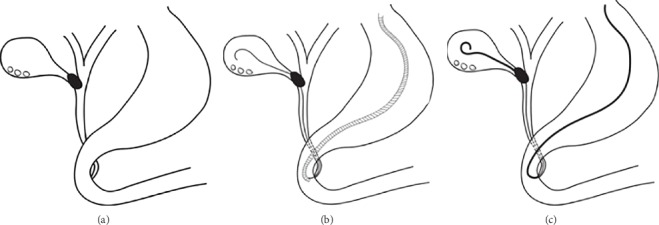
The procedure of endoscopic nasogallbladder drainage (ENGBD). (a) Cholangiography for diagnosis through endoscopic retrograde cholangiopancreatography (ERCP). (b) Using a hydrophilic guidewire under fluoroscopy, the catheter can be inserted into the target bile duct and then the gallbladder. The guidewire was retained in the gallbladder cavity to the greatest extent possible, in order to form a circle. (c) The curved nasobiliary duct was pushed into the gallbladder along the guidewire; the guidewire was then gently removed. The head of the nasobiliary duct thus formed a circle in the gallbladder cavity.

All four patients underwent ENGBD. First, ERCP intubation and cholangiography were performed for the diagnosis of patients with high risk for type I Mirizzi syndrome. In the case of common bile duct stones, stones were extracted using a stone extraction basket or balloon, in order to clear the biliary tract. Duodenal papillotomy was performed when necessary. Then, after successful intraoperative intubation during ERCP, under fluoroscopy with the use of a hydrophilic guidewire, a catheter was inserted into the cystic duct, then the gallbladder, as much of the guidewire as possible was retained in the gallbladder cavity. Next, under fluoroscopy, the curved nasobiliary duct was pushed into the gallbladder along the guidewire. The guidewire was then gently removed. The head of the nasobiliary duct thus formed a circle in the gallbladder cavity (Figure 1(a)). After a syringe was successfully positioned in the nasobiliary duct, it was used to extract as much bile as possible from the gallbladder as far as possible to decompress the gallbladder, and the bile extracted can be used for bacterial culture. Drainage during ENGBD successfully relieved the patients' symptoms of acute cholangitis and cholecystitis (Figure 1(b)). The second phase of laparoscopic treatment was performed via laparoscopic cholecystectomy, bile duct exploration, or T-tube drainage, as dictated by the specifics of the patient's condition (Figure 2).

## 3. Results

**Figure 3:**
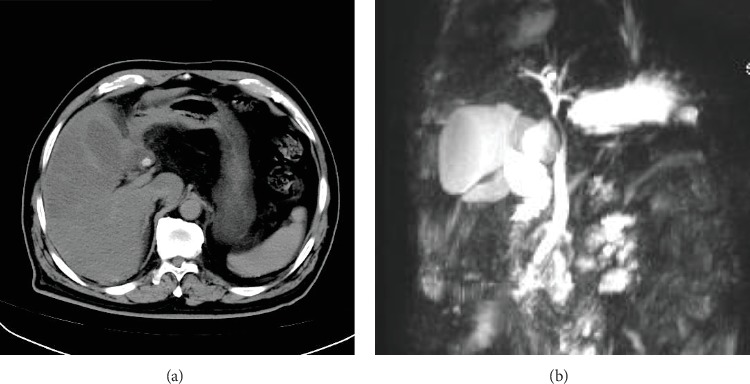
Preoperative imaging of Case 2. (a) A computerized tomography (CT) scan shows gallbladder enlargement and dilation of the intrahepatic bile duct. (b) Magnetic resonance cholangiopancreatography (MRCP) imaging shows external compression of the common bile duct by a cystic duct stone.

Three male patients and one female patient, ranging in age from 35 to 72 years old, were included in the study. All four patients were admitted to the emergency department at the hospital. Three patients had initial symptoms of abdominal pain and jaundice, and one patient presented with fever, chills, and jaundice (Table 2). After obtaining a detailed medical history from each patient, we found that three patients had a clear history of gallstone disease; the fourth patient had a history of upper right abdominal pain without systemic examination (Table 1). Laboratory blood testing revealed elevated levels of white blood cells and bilirubin in all four patients, although the magnitude of the elevation varied from patient to patient. CT revealed enlarged gallbladders in all 4 patients. This finding is consistent with acute cholecystitis. Dilation of the intrahepatic bile duct was observed in Cases 1 and 2 (Figure 3(a)). Multiple gallstones, as well as intra- and extrahepatic bile duct dilation were observed in Cases 3 and 4. In Cases 1, 2, and 3, the results of MRCP indicated that the presence of a cystic duct stone was compressing the common bile duct (Figure 3(b)). This finding was consistent with Mirizzi syndrome. In Case 4, MRCP revealed structural abnormalities in Calot's triangle.

Based on the findings collected preoperatively, including medical history and the results of physical and adjuvant examinations, Mirizzi syndrome was highly suspected in all four patients. Cases 3 and 4 had concurrent choledocholithiasis. The detailed preoperative diagnoses can be found in Table 1.

All four patients were quickly given antibiotics, fluid replacement, and symptomatic treatment then prepared for emergency surgery after admission. Considering that all patients had acute cholecystitis and cholangitis, we decided first to perform ERCP. All four patients included in the study had been diagnosed with type I Mirizzi syndrome with acute cholecystitis. In each case, the diagnosis had been confirmed by ERCP examination. Because obstruction of the cystic duct had led to acute cholecystitis in these patients, intraoperative ENGBD was performed. Stones were found in the common bile duct in Cases 3 and 4. In Case 3, endoscopic sphincterotomy (EST) and endoscopic papillary balloon dilation (EPBD) were performed simultaneously to remove the bile duct stones. In Case 4, the patient underwent ENGBD only. No attempt was made to remove the bile duct calculi because of the large size of the stones and the bile duct edema caused by inflammation. The clinical symptoms of Case 4, which included abdominal pain, fever, and jaundice, were significantly relieved after ENGBD treatment.

After 2–5 days of recovery, all four patients underwent elective laparoscopic surgery. Cases 1, 2, and 3 underwent laparoscopic cholecystectomy (LC), while Case 4 underwent laparoscopic cholecystectomy and laparoscopic common bile duct exploration (LCBDE). The ENGBD tubes of all 4 patients were removed during surgery. A summary of the surgical plan is presented in Table 1.

None of the four patients experienced a severe postoperative complication. Case 4 had a minor amount of bile leakage, which was cured by ultrasound-guided abdominal puncture drainage. The T-tube was successfully removed after the results of T-tube angiography at the three-month postoperative follow-up visit confirmed the absence of any residual stones.

All patients were followed up for 12 months. No stone recurrence or related complication was noted in any case, indicating excellent short-term outcomes of this surgical approach.

## 4. Discussion

Because of the difficulty of preoperative diagnosis and the complexity of pathological changes in patients with Mirizzi syndrome, laparoscopic treatment for the disease is difficult and is associated with high risk for complications (4, 5). It has been reported that patients with Mirizzi syndrome have a high rate of conversion from LC to open cholecystectomy (1). Recently, ERCP has become the first-line method for the diagnosis of Mirizzi syndrome, with accuracy as high as 90% (6, 7). ERCP is helpful for the Csendes classification, as the use of this technique can more clearly identify bile duct compression, as well as the presence of a bile duct gallbladder fistula or a gallbladder intestinal fistula. In the cases presented here, the diagnosis of type I Mirizzi syndrome with acute cholecystitis was confirmed by ERCP examination.

At present, first-stage ERCP and ENBD combined with second-stage surgery have become the preferred treatment for all types of Mirizzi syndrome. This is because ENBD can be performed expediently to remove the biliary obstruction and guide the shape of the common bile duct during second-stage surgery. The combination of ENBD with laparoscopic surgery is reported to be a safe and effective treatment for Mirizzi syndrome. Compared with other surgical approaches, the combination of ENBD with laparoscopic surgery results in less trauma, less pain, and faster recovery times (8). However, in clinical practice, we found that patients with Mirizzi syndrome often had varying degrees of accompanying acute cholecystitis. Because stones and inflammation in the gallbladder neck could not only compress the hepatic duct but also contribute to gallbladder duct obstruction, the results of imaging examinations in patients with Mirizzi syndrome show enlargement of the gallbladder accompanied by exudation. In such cases, ENBD may only solve the problem of biliary obstruction, with no relief of the associated inflammation. Severe complications such as gallbladder perforation can also occur after ENBD treatment (9). The physicians in the Department of Hepatobiliary and Pancreatic Surgery at the First Hospital of Jilin University have improved upon existing technology for the treatment of type I Mirizzi syndrome and acute cholecystitis through the combination of ENGBD with laparoscopic surgery. As the results presented above for four cases demonstrate, this modified treatment has proven to be effective for patients with type I Mirizzi syndrome and acute cholecystitis.

ENGBD has a history of more than 30 years. Kozarek (10) completed ENGBD for the first time in 1984. Foerster and his colleagues (11) performed a detailed inspection of the gallbladder with ENGBD in 1988. In 1991, Tamada et al. (12) applied ENGBD to the treatment of acute cholecystitis. Similar reports were followed by Johlin and Neil (13) in 1993 and Nakatsu et al. (14) in 1997. With the improvement of the cannula and guidewire and the development of endoscopy technology, the indications of ENGBD continue to expand. Since 1995, Arisaka et al. (15) have proactively performed ENGBD in acute cholecystitis and Mirizzi's syndrome patients who have a bleeding tendency or suspected complicated gallbladder cancer, and achieved good clinical results. A clinical study by Toyota et al. (16) in 2006 indicated ENGBD could alleviate inflammation and fix operator's aim during early laparoscopic cholecystectomy in the treatment of acute cholecystitis.

According to these reports, the success rate of ENGBD was 53.8-90.0% (5, 13, 15, 16). An investigation reported by Masuda et al. (17) revealed that the success rate of ENGBD in patients with no acute inflammation was 75.6% (90/119) and 71.0% (22/31) in patients with acute cholecystitis and Mirizzi syndrome, which was slightly low. Arisaka et al. (15) reported that they were able to perform ENGBD in 62.5% (15/24) of acute cholecystitis patients and in 100% (7/7) of patients with Mirizzi syndrome successfully. In our study, we successfully performed ENGBD in all four patients and achieved satisfactory drainage effect. Although the success rate is high, it does not mean the technology is simple. In Mirizzi syndrome patients with acute cholecystitis, occlusions due to stones and cancer and edema due to inflammation increase the difficulty of ENGBD. In addition, the success rate of ENGBD is significantly related to the branching form of the cystic duct. Kanemaki et al. (18) reported that the success rate in the patients with parallel type of the cystic duct was higher than that in the patients with other branching forms (angular and spiral types) of the cystic duct. Therefore, ENGBD might not be achieved for the patients with occluded cystic duct due to severe stone or tumor impaction, or the patients with angular or spiral types of the cystic duct.

Nuzzo et al. (19) reported that the incidence of bile duct injury associated with LC is 0.4-0.6%. The rate is higher in patients with acute cholecystitis or Mirizzi syndrome. This is because it is difficult to anatomically distinguish inflammatory adhesions within Calot's triangle in these patients, which would make identification of the cystic duct problematic, and raise the risk of biliary or vascular injury. Although ENBD is superior to ENGBD in identifying the common bile duct, the identification and operation of the cystic duct is a key step in following LC or LCBDE. With the help of ENGBD, surgeons can clearly distinguish the cystic duct and its direction in anatomy, which greatly improve the safety and success rate of the operation.

The ENGBD-laparoscopic approach has the following advantages. First, this technique integrates diagnostic and treatment approaches. ERCP is the preferred option for diagnosing Mirizzi syndrome. When performed promptly after the definitive diagnosis, ENGBD can enhance the effectiveness of the treatment. Second, this approach allows for precise drainage. Traditional ENBD removes pus and bile only from the common bile duct, without addressing concurrent acute cholecystitis caused by cystic duct obstruction. In severe cases, patients have symptoms such as abdominal pain and fever after the operation, in addition to complications such as gallbladder perforation. The ENGBD utilizes the multilateral hole in the nasal bile duct to sufficiently drain the gallbladder cavity and bile duct (20). Last but not least, this approach improves surgical safety. Traditional surgery, especially laparoscopic surgery, is risky for the treatment of Mirizzi syndrome. Because the anatomical structure varies greatly from patient to patient, complications such as biliary tract injury are common. ENGBD may not only relieve inflammation around the gallbladder triangle but also facilitate the confirmation of anatomical location. ENGBD can also be used as a guide to avoiding complications such as biliary tract injury (21).

In the clinical study reported by Arisaka et al. (15), jaundice persisted in two patients with Mirizzi's syndrome, and it required the addition of endoscopic nasobiliary drainage. This might be due to the flow of infected bile into the lumen of the common bile duct, or poor drainage of the bile duct due to the narrowing of the papilla caused by the placement of ENGBD. In our study, the jaundice of all patients was relieved after ENGBD. This was because we used the nasobiliary duct with multiple side holes, which could not only lead out the bile in the gallbladder but also drain the bile from the common bile duct. In addition, we performed EST or EPBD for the patients with narrow papilla or large bile duct stones to reduce postoperative complications.

The other complications of ENGBD include acute pancreatitis and penetration of the cystic duct which is because inflammatory edema could cause the wall of the cystic duct to become weak and so caution is required (15). Although there are still some problems to be solved, the complication rate of ENGBD is relatively low. Therefore, it is considered that ENGBD is useful for Mirizzi's syndrome with acute cholecystitis before the following laparoscopic surgery.

## 5. Conclusions

The combination of ENGBD and laparoscopic surgery for type I Mirizzi syndrome with acute cholecystitis is safe and feasible. This approach can reduce the traumatic stress to patients and is worthy of widespread implementation. As the number of cases remains limited, we will continue to accumulate data in order to further evaluate the efficacy of this approach in the treatment of other manifestations of Mirizzi syndrome.

## Figures and Tables

**Table 1:** Clinical diagnosis and treatment data for the four patients with type I Mirizzi syndrome included in the study.

Clinical information	Case 1	Case 2	Case 3	Case 4
Medical history	History of gallbladder stones	History of upper right abdominal pain without systemic examination	History of gallbladder stones	History of gallbladder stones
Preoperative consultation (yes or no)	Yes	Yes	Yes	Yes
Operation plan	ENGBD+LC	ENGBD+LC	EST+EPBD and stone removal+ENGBD+LC	ENGBD+LCBDE
Surgical interval (days)	2	2	5	4
Recovery of operation	Very well	Very well	Very well	A small amount of bile leakage occurred after the operation but cured by ultrasound-guided puncture drainage. Three months later, returned to the hospital to pull out the T-tube
Length of hospitalization (days)	12	11	13	16

Abbreviation: ENGBD: endoscopic nasogallbladder drainage; LC: laparoscopic cholecystectomy; EST: endoscopic sphincterotomy; EPBD: endoscopically performed biliary drainage; LCBDE: laparoscopic common bile duct exploration.

**Table 2:** The clinical characteristics for the four patients with type I Mirizzi syndrome included in the study.

Characteristics	Case 1	Case 2	Case 3	Case 4
Age (years)	46	35	66	72
Gender	Male	Male	Female	Male
Initial symptom	Abdominal pain and jaundice	Fever, chills, and jaundice	Abdominal pain and jaundice	Abdominal pain and jaundice
Imagological examination	CT: gallbladder enlargement and intrahepatic bile duct dilatation	CT: gallbladder enlargement and intrahepatic bile duct slight dilatation	CT: gallbladder enlargement, gallbladder stones, and intrahepatic and extrahepatic bile duct dilation	CT: gallbladder enlargement, gallbladder stones, and intrahepatic and extrahepatic bile duct dilation
	MRCP: the cystic duct stone causing extrinsic compression of the common bile duct	MRCP: the cystic duct stone causing extrinsic compression of the common bile duct	MRCP: the cystic duct stone causing extrinsic compression of the common bile duct and common bile duct stone	MRCP: disordered structure relationship at the gallbladder triangle area and a stone at the end of the common bile duct
Leukocyte (×10^9^/L)	13.5	21.9	17.9	12.7
Total bilirubin (*μ*mol/L)	75.5	65.8	40.9	111.2
Direct bilirubin (*μ*mol/L)	44.8	47.0	28.9	60.7

Abbreviation: CT: computerized tomography; MRCP: magnetic resonance cholangiopancreatography.

## Data Availability

The datasets generated and analyzed during the present study are available from the corresponding author on reasonable request.
